# The Use of Carbon Nanotubes to Reinforce 45S5 Bioglass-Based Scaffolds for Tissue Engineering Applications

**DOI:** 10.1155/2013/465086

**Published:** 2013-11-04

**Authors:** R. Touri, F. Moztarzadeh, Z. Sadeghian, D. Bizari, M. Tahriri, M. Mozafari

**Affiliations:** ^1^Biomaterial Group, Faculty of Biomedical Engineering (Center of Excellence), Amirkabir University of Technology, P.O. Box 15875-4413, Tehran, Iran; ^2^Department of Gas, Research Institute of Petroleum Industry (RIPI), P.O. Box 14857-3311, West Boulevard, Azadi Sport Complex, Tehran, Iran; ^3^Bioengineering Research Group, Nanotechnology and Advanced Materials Department, Materials and Energy Research Center (MERC), P.O. Box 14155-4777, Tehran, Iran

## Abstract

Bioglass has been used for bone-filling material in bone tissue engineering, but its lean mechanical strength limits its applications in load-bearing positions. Carbon nanotubes (CNTs), with their high aspect ratio and excellent mechanical properties, have the potential to strengthen and toughen bioactive glass material without offsetting its bioactivity. Therefore, in this research, multiwall carbon nanotube (MWCNT)/45S5 Bioglass composite scaffolds have been successfully prepared by means of freeze casting process. 45S5 Bioglass was synthesized by the sol-gel processing method. The obtained material was characterized with X-ray powder diffraction (XRD). The mechanical properties of the scaffolds, such as compression strength and elastic modulus, were measured. Finally, compared with the scaffolds prepared by 100% 45S5 Bioglass powders, the addition of 0.25 wt.% MWCNTs increases the compressive strength and elastic modulus of 45S5 Bioglass scaffolds from 2.08 to 4.56 MPa (a 119% increase) and 111.50 to 266.59 MPa (a 139% increase), respectively.

## 1. Introduction

Tissue engineering is a concept that promotes the regeneration of host tissue by designing the scaffold that is populated with cells and signaling molecules. The scaffold is a three-dimensional substrate that can act as a template for tissue regeneration. The specific properties of ideal scaffolds for bone tissue engineering can be defined as good biocompatibility, optimal porous structure with pore interconnectivity, and ability to deliver cells. In addition, the scaffolds should possess appropriate mechanical strength and biodegradation rate without any undesirable by-products [1].

Carbon nanotubes (CNTs) are nanosized cylindrical carbon tubes with very large aspect ratios. CNTs can be categorized as (i) single-walled carbon nanotubes (SWCNTs) and (ii) multiwalled carbon nanotubes (MWCNTs) [2]. SWCNTs are constructed of single sheets of graphite diameters ranging from 0.4 to 2 nm, while MWCNTs consist of multiple concentric graphite cylinders with increasing diameters ranging from 2 to 100 nm [3, 4].

These materials have excellent characteristics that make them potentially useful in many applications such as biomaterials science and scaffolding for bone regeneration. Bone tissue compatibility of CNTs gives them an influential role on the bone formation. Also, applying CNTs in synthetic bone materials can improve their overall mechanical properties, and they act as appropriate scaffolds to promote and guide bone tissue regeneration [5].

CNTs have been extremely applied as reinforcing fibers in polymer and metals matrix composites [6–10], and also, several recent experiments on the preparation and mechanical properties of CNT-reinforced ceramic-matrix composites have been reported [11–17].

Mattioli-Belmonte et al. [18] have recently reported the fabrication of MWCNTs-polycaprolactone composites, in which both the quantity of nanotubes in the matrix as well as the scaffold design were varied in order to tune the characteristics of the scaffolds. They reported that by changing the ratio of MWCNTs, the elastic modulus of the nanocomposites, osteoblast proliferation, and modulate cell morphology could be adjusted for bone tissue engineering applications.

Bioactive glasses are predestined materials to develop suitable materials for medical applications such as using as implants in the human body to repair and replace diseased or damaged bone due to their excellent bioactivity as well as biocompatibility. However, contrary to their outstanding bioactivity characteristics, these biomaterials exhibit low mechanical strength such as fracture toughness in comparison with natural bone [19]. Therefore, in order to improve the mechanical behavior of bioactive glass materials, they are always reinforced with other materials, such as polymer and fiber [20].

Among all kinds of bioactive glasses, 45S5 Bioglass has been used in a number of medical devices due to its approval by the U.S. Food and Drug Administration (FDA). In 1969, Hench and his colleagues developed 45S5 Bioglass with the nominal composition of 46.14 mol% SiO_2_, 24.35 mol% Na_2_O, 26.91 mol% CaO, and 2.60 mol% P_2_O_5_. It has been successfully used in orthopedic and dental surgery [20, 21].

Commercially produced bioactive glasses have been made by conventional glass powder manufacturing methods, that is, melting and quenching. Meanwhile, increasing research efforts are being invested in fabrication of bioactive glasses using the sol-gel technique, due to its advantages over melting-quenching processes [22]. Sol-gel processing involves the synthesis of a sol followed by the formation of a gel by chemical reaction or aggregation and lastly thermal treatment for drying, organic removal, and sometimes crystallization. Compared with conventional melt-processed BGs, sol-gel BGs are processed at lower temperatures and have better compositional control [23]. Sol-gel-derived bioactive glasses exhibit high specific area, high osteoconductive properties, and also a significant degradability [24].

Freeze casting, as an effective method for preparation of porous structures, was seen a great deal of efforts in recent years. This method includes freezing a liquid suspension, followed by sublimation of the solidified phase, and subsequent sintering to densify the walls, resulting in a porous structure with unidirectional pores in the case of unidirectional freezing, where pores are a replica of the solvent crystals [25]. The microstructure of products is lamellar, with long parallel pores aligned in the movement direction of the ice front [26]. The main goal of this research is to evaluate the effect of CNT on the mechanical properties of freeze-casted CNT/45S5 Bioglass composites as a scaffold for bone tissue engineering.

## 2. Experimental Procedure

### 2.1. Materials

The chemicals used in this research include tetraethyl orthosilicate (TEOS: C_8_H_20_O_4_Si; Merck Co.), calcium nitrate tetrahydrate (Ca(NO_3_)_2_·4H_2_O; Merck Co.), sodium nitrate (NaNO_3_; Scharlau Co.), triethyl phosphate (TEP: C_6_H_15_O_4_P; Merck Co.) and nitric acid (HNO_3_; Merck Co.) for the synthesis of the sol-gel 45S5 bioactive glass, and polyvinyl alcohol (PVA; Merck Co.) and carboxymethyl cellulose (CMC; Fluka biochemika Co.) as additives for slurry preparation, and multiwalled carbon nanotubes (MWCNT; supplied by Research Institute of Petroleum Industry) as a reinforcement phase were used for the composite preparation.

### 2.2. Synthesis of Multiwalled Carbon Nanotubes

Multiwalled carbon nanotubes (MWCNTs) were prepared by spray pyrolysis, a type of catalytic chemical vapor deposition (CCVD) method, in which the carbon source, in the form of liquid hydrocarbons, acts as a solvent for the catalyst and is sprayed into the furnace. Ferrocene was used as a catalyst precursor, and hexane, a good solvent for ferrocene, was used as a carbon source. The sublimation temperature of ferrocene, the ferrocene concentration in hexane, pyrolysis temperature and time, and the flow rate of hexane and H_2_ were optimized to obtain MWCNTs with a high quality and a high yield. For the same of removing the impurities, MWCNTs were purified with acid leaching and air oxidation. The MWCNTs have a typical sausage-like structure and their lengths are more than several tens of micrometers. The inner and outer diameters of the MWCNTs were in the range of 15–45 and 25–70 nm, respectively. The purity and yield of the purified MWCNTs were more than 95% and 70% mass fraction, respectively [27].

### 2.3. Synthesis of Bioactive Glass

The molar ratios of TEOS, TEP, NaNO_3_, and Ca(NO_3_)_2_·4H_2_O were designed according to the molar ratios of SiO_2_, P_2_O_5_, Na_2_O, and CaO in 45S5. In the first step, the solution was prepared as follows: 41.2312 g (0.19792 mol) of TEOS was added into 16 mL of 1 M nitric acid; the mixing process was allowed to be continued for at least 1 h for the acid hydrolysis of TEOS. In the second step, each chemical in the sequence was added reasonably slowly into the aqueous solution at room temperature only when the previous solution became clear, and was then stirred for at least 1 h. The following reagents were added in sequence: 4.3420 g (0.0238 mol) TEP, 13.8925 g (0.1635 mol) of sodium nitrate, and 26.7423 g (0.1132 mol) of calcium nitrate tetrahydrate. The next step is to stop stirring and leave the above sol to gel at the ambient condition. The resulting gel was dried at 60 and 200°C for 72 and 40 h, respectively, aged at 600°C for 5 h and sintered at 1000°C for 2 h. The main purpose of sintering the aged gel was to decompose sodium nitrate NaNO_3_ and calcium nitrate Ca(NO_3_)_2_ in order to obtain Na_2_O and CaO in the material. The full thermal decomposition of NaNO_3_ and Ca(NO_3_)_2_ occurs at about 680°C and 560°C, respectively, and the crystallization temperature of 45S5 Bioglass is 600°C. Hence, crystallization occurs in the glasses during the decomposition treatment of NaNO_3_ and Ca(NO_3_)_2_. The crystallization, however, would not be problematical provided that the crystalline phase is Na_2_Ca_2_Si_3_O_9_ with the chemical composition of 50 mol% SiO_2_, 16.67 mol% Na_2_O, and 33.33 wt.% CaO, which couples well mechanical strength with appropriate biodegradability [21]. To ensure this, the sintering condition was set at 1000°C in order to achieve both suitable mechanical properties and biodegradability of the material [28]. Sintered powders were then milled in a planetary milling machine (model: SV-iG5, 0.75–3.7 kW, 200 V, rpm: 300) for 30 minutes with alumina balls to eliminate the agglomeration and reduce the particle size of bioactive glass powders. The average particle size of the powder is less than 1 *μ*m.

### 2.4. Scaffold Fabrication

**Figure 1:**
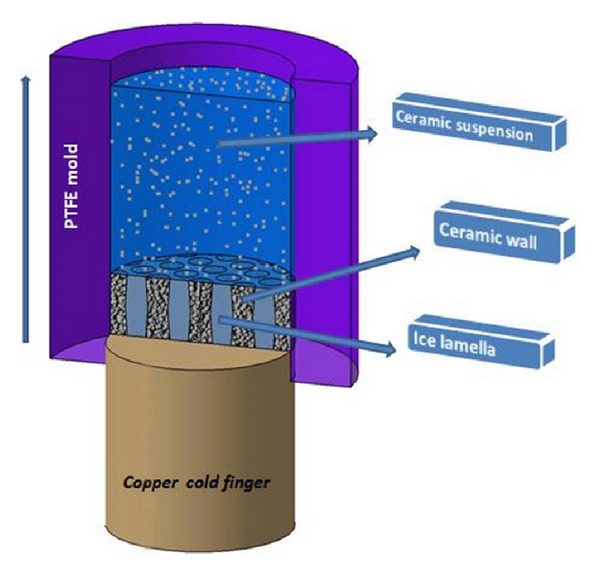
A schematic of freeze casting technique for the fabrication of the MWCNT/45S5 Bioglass scaffolds.

For composite preparation, the synthesized 45S5 Bioglass powders were reinforced with different weight fractions of MWCNTs. The porous inorganic scaffolds were prepared by controlled freezing of CNT/45S5 Bioglass slurries. Before, for purifying and stabilizing of MWCNTs, these powders were refluxed in a mixture of oxidizing acids which include sulfuric and nitric acid (with the volume ratio of 3 : 1 of sulfuric to nitric acid), for 4 h to oxidize and remove the metal catalysts and carbonaceous deposits from the inside and outside of the tube. Slurries were prepared by mixing distilled water with an organic binder such as PVA (equal to 1 wt.% of bioactive glass powder weight), a dispersant such as CMC (2 wt.% of bioactive glass), the MWCNT powder in different ratios (0, 0.1, 0.25, and 0.5 wt.% of bioactive glass, resp.), and the bioactive glass powder (20 vol.% of the slurry). The slurries were ball-milled for 24 h with alumina balls to break the agglomerated particles, and then deaired by stirring in a vacuum desiccator. To improve homogeneity and prevent the agglomeration, MWCNTs were ultrasounded for 2 h. Freezing of the slurries was done by pouring them into a PTFE mold, connected to a copper cold finger, which is placed in liquid nitrogen. With temperature decrease, the thermal energy is mainly transmitted in one direction due to the thermal conductivity coefficient of the slurry being higher than that of PTFE; therefore, the ice grows in one direction, resulting in the unidirectional microstructure of the ceramics [29].  Figure 1 shows that the system consists of the ice lamellae, the ceramic walls, and the liquid particle suspension. The growing ice lamellae are pushing the ceramic particles into the interlamellar spaces, where they form ceramic walls consisting of random close packed ceramic particles dispersed in ice. In other words, the ice expels the ceramic particles as it grows and leaves a layered ceramic structure after water removal [30, 31]. Frozen samples were freeze-dried (Freeze dryer CHRIST, ALPHA 1-2 LD, Germany) at low temperature (−60°C, temperature of the cold finger of the freeze dryer) and low pressure (1.3 kPa) for 72 h. Sintering of the green bodies was done in a tube box furnace containing argon atmosphere in 900°C for 3 h, with heating and cooling rates of 10°C/min.

### 2.5. Characterization

#### 2.5.1. X-Ray Diffraction

The resulting 45S5 sol-gel derived Bioglass powders were analyzed by X-ray diffraction (XRD) with Philips PW 1800 diffractometer. This instrument works with voltage and current settings of 40 kV and 30 mA, respectively, and uses Cu-K*α* radiation (1.5405 Å). For qualitative analysis, XRD diagrams were recorded in the interval 4° ≤ 2*θ* ≤ 90° at scan speed of 2°/min.

#### 2.5.2. Scanning Electron Microscopy (SEM)

The samples were coated with a thin layer of gold (Au) by sputtering, and then, the microstructure of the scaffolds was observed on a scanning electron microscope (SERON Technologies Company, AIS2100) that operated at the acceleration voltage of 20 kV.

#### 2.5.3. Mechanical Properties

The compression test was carried out on a testing machine (Zwick/Roell) dynamic testing machine (DTM) model, Hct 400/25, Germany, with a crosshead speed of 1 mm/min according to ASTM F-2150 (load cell: 25 kN; resolution: 1 N). The samples were cylindrical in shape, with dimensions 20 mm in height and 12 mm in diameter. Each test has been repeated five times and the average amount and standard deviation (SD) of related parameters were determined.

#### 2.5.4. Scaffolds' Shrinkage and Porosity Measurement

**Figure 2:**
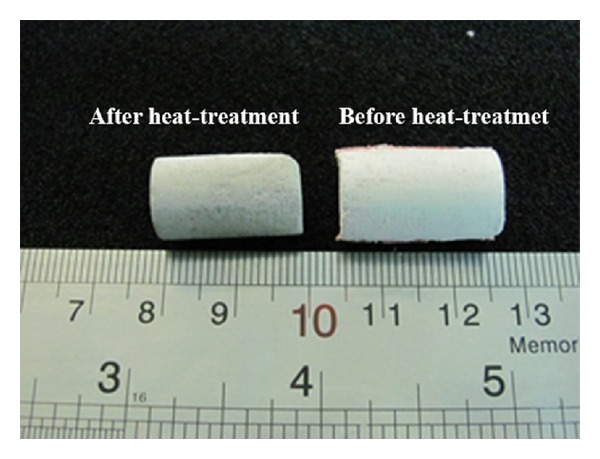
An optical image of the fabricated 0.5 wt.% MWCNT/45S5 Bioglass composite scaffolds before and after heat treatment at 900°C, which shows the shrinkage of samples.

The samples' shrinkage was calculated at specific temperatures from the variation of the samples' area, using (1), which assumes isotropic shrinkage [32]:

(1) $$\begin{array}{c} \text{Shrinkage}\left(\%\right)=\frac{A_{0}-A_{T}}{A_{0}}\times 100, \end{array}$$

where *A*_0_ = the initial area of the specimen at room temperature, and *A*_*T*_ = the area of the specimen at temperature *T* = 900°C. The calculated shrinkage of samples is around 13.5%, as shown in Figure 2.

The density of the scaffolds (*ρ*_foam_) was determined from the mass and dimensions of the sintered bodies. The porosity (*p*) was then calculated by (2)

(2) $$\begin{array}{c} P=1-\left(\frac{\rho_{\text{foam}}}{\rho_{\text{solid}}}\right)=1-\rho_{\text{relative}}, \end{array}$$

where *ρ*_solid_ = 2.7 g/cm^3^ is the density of composite calculated by the rule of mixtures [28].

All scaffolds exhibited porosity of ~63%, as determined by measurement of their mass and dimensions and applying (2). The measured porosity percentage is in the range of natural spongy bone (30–90%) [33].

### 2.6. Statistical Analysis

All experiments were performed in fifth replicate. The results were given as means ± standard error (SE). Statistical analysis was performed by using One-way ANOVA and Tukey test with significance reported when *P* < 0.05. Also, for investigation of group normalizing, Kolmogorov-Smirnov test was used.

## 3. Results and Discussion

### 3.1. Microstructural Analysis

**Figure 3:**
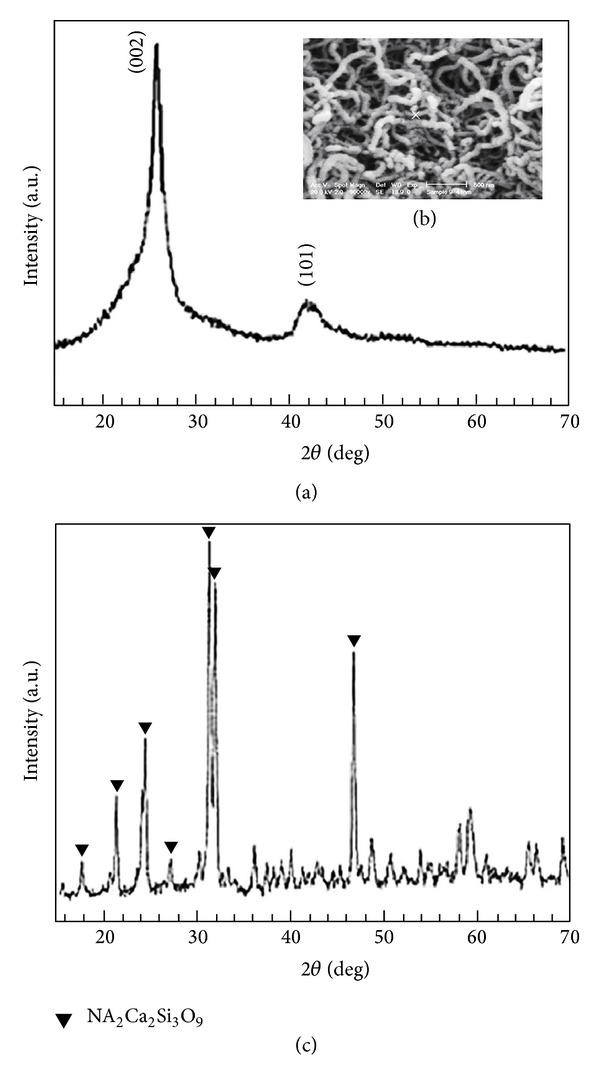
(a) XRD spectra of the synthesized MWCNTs, (b) SEM micrograph of the synthesized MWCNTs, and (c) XRD spectra of the sol-gel derived 45S5 Bioglass after sintering at 1000°C for 2 h.

Figure 3(a) shows the XRD pattern of the purified CNTs sample. The peaks were indexed as (002) and (101) reflections of hexagonal graphite. The peaks corresponding to the catalytic impurities (Fe) have not been found for the purified samples. The *d*_002_ value, corresponding to the (002) peak (2*θ* = 26.5°), was evaluated to be 0.342 nm, which was very close to that of graphite (0.335 nm) [34]. The presence of (002) peak in the XRD pattern suggested that the CNTs were MWCNTs. The average crystallite sizes *D* were determined according to the Debye-Scherrer formula (*D* = *kλ*/*β*cos⁡*θ*, where *k* is a constant (shape factor, about 0.9), *λ* is the X-ray wavelength (1.5405 Å as mentioned before), *β* is the full width at half maximum (FWHM) of the diffraction line, and *θ* is the diffraction angle). Based on the full width at half maximum of the reflection from (101) peak at 2*θ* = 26.5°, the mean crystallite sizes of MWCNTs were estimated approximately as 35 nm.

It is always difficult to measure the accurate length of the MWCNTs from the SEM observation because of their twisting morphology, but the length can be estimated to be more than several tens of micrometers. As shown by the SEM image of the purified MWCNTs in Figure 3(b), the catalytic particles and amorphous carbon were removed successfully by the purification, and apart from that it did not cause any damage to the MWCNTs. 

Figure 3(c) shows the XRD pattern of the sol-gel-derived 45S5 Bioglass powder. The crystalline phase Na_2_Ca_2_Si_3_O_9_ was identified in the as-sintered 45S5 Bioglass powders. It is notable that 45S5 Bioglass could not be completely crystallized. From the components of 45S5 Bioglass and Na_2_Ca_2_Si_3_O_9_, it can be seen that the Na_2_Ca_2_Si_3_O_9_ phase would require too much CaO to fully crystallize from Bioglass. Eventually, CaO is depleted when the crystallinity reaches 80.7 mol.% (i.e., 77.4 wt.%), which is the maximum crystallinity achievable with the 45S5 Bioglass composition [28].

### 3.2. Compression Test

In a recent experiment, Xu et al. [35] demonstrated the potential use of MWCNTs to overcome the brittleness of hydroxyapatite (HA) constructs. Though they have not detected any obvious chemical reactions between MWCNTs and HA nanoparticles, they have reported a relatively high value of modulus (~131.1 GPa) and hardness (~6.86 GPa). Moreover, the in vitro cellular responses to the composites showed relatively acceptable results in contact with human osteoblast cell line.

**Figure 4:**
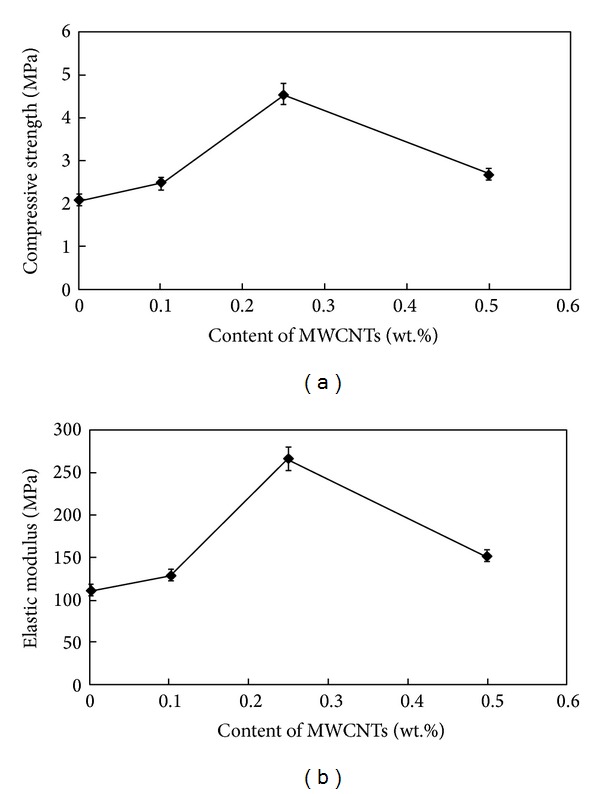
(a) Compressive strength and (b) elastic modulus of MWCNTs/45S5 Bioglass composite scaffolds as a function of MWCNTs content.

Figure 4(a) shows the relationship between the compressive strength of scaffolds and the weight fraction of MWCNTs, indicating that the compressive strength of scaffolds increases with the increase in weight fraction of MWCNTs from 0.1 to 0.5 wt.%. The addition of 0.25 wt.% MWCNTs increases the compressive strength of 45S5 Bioglass scaffolds from 2.08 to 4.56 MPa (a 119% increase). This increase is due to good MWCNT/45S5 Bioglass interfacial bonding, and the MWCNTs are dispersed well within the 45S5 Bioglass matrix. However, the strengthening effect of MWCNTs decreases with a further increase in the MWCNT weight fraction to 0.5 wt.%, as the compressive strength decreases from 4.56 to 2.7 MPa. However, it is still higher than the pure 45S5 Bioglass sintered part. The decrease is mainly attributed to the nanocomposite scaffolds' lower relative density due to the agglomeration of additional MWCNTs, which have a lower density than the glass matrix. The agglomeration of MWCNTs weakens the bonding between the CNTs and the Bioglass. 

In a recent study, Nezafati et al. [36] reported an almost similar response after adding a high amount of second phase to the matrix. After analyzing the effect of structure and amount of BGF incorporation into the CPC system, and the effect of mechanical compaction on the glass fiber-modified systems calcium phosphate cements, they reported that the compressive strength of the set cements without any fibers was 0.635 MPa, which was optimally increased to 3.69 MPa by applying 15% fibers and then decreased by further addition of it. In addition, both the work-of-fracture and elastic modulus of the cement were considerably increased after applying the fibers in the cement composition. 

Figure 4(b) shows the relationship between the elastic modulus of scaffolds and the weight fraction of MWCNTs. The elastic modulus initially increases with increasing the weight fraction of MWCNTs until 0.25 wt.%; above this value, the elastic modulus decreases reaching values close to that of 45S5 Bioglass scaffold. The elastic modulus of the scaffolds shows a similar trend to the compressive strength. The addition of 0.25 wt.% MWCNTs increases the elastic modulus of 45S5 Bioglass scaffold from 111.50 to 266.59 MPa (a 139% increase). This increase is due to the dispersion of MWCNTs in the matrix, which serves as a reinforcing phase. However, with a further increase in the MWCNT weight fraction to 0.5 wt.%, the elastic modulus decreases from 266.59 to 151.88 MPa. This behavior could be due to the electrostatic nature of CNT. In fact, if CNT concentrations are too high, the repulsive forces present on their surface could induce an inhomogeneous dispersion of composite materials, leading to an abrupt decrease of elastic modulus as well as an increase in fragility [18].

 Jia et al. [20] evaluated the effect of MWCNT content on the mechanical properties of MWCNT/45S5 Bioglass bulk composites, in order to identify the optimal processing parameters. They discussed that brittle materials could be strengthened and toughened by the addition of the second phase materials such as fibers, provided that crack deflection occurs at the interfaces between the fibers and the matrix. They reported that the MWCNTs were homogeneously dispersed within the 45S5 Bioglass matrix in the 5 wt.% MWCNT/45S5 Bioglass composites and that the presence of an ideal MWCNT/45S5 Bioglass interfacial structure is suitable for crack deflection mechanism, but the MWCNTs did not disperse uniformly within the 45S5 Bioglass matrix in the composites with 9 wt.% MWCNTs, which resulted in a lower relative density of the composites. Hence, flexural strength and fracture toughness are lower than those with the weight fraction of 5 wt.%.

**Figure 5:**
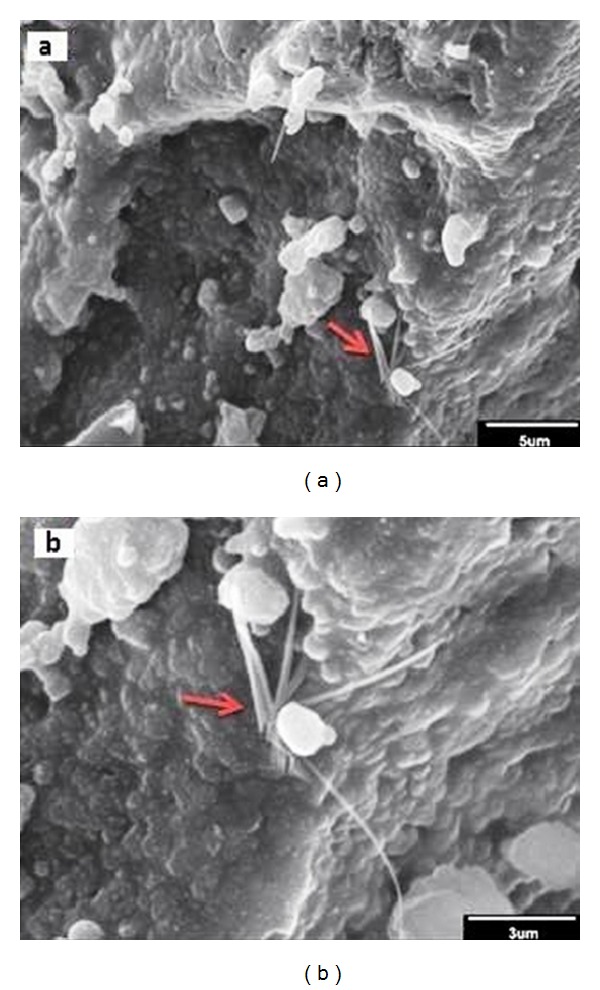
(a) Low magnification and (b) high magnification SEM micrographs of the agglomerated MWCNTs in the scaffolds with 0.5 wt.% MWCNTs.

Figure 5 shows SEM images of agglomerated MWCNTs in the scaffolds with 0.5 wt.% MWCNTs. It can be observed that the MWCNTs did not disperse uniformly within the 45S5 Bioglass matrix, so compressive strength and elastic modulus are lower than scaffolds with the weight fraction of 0.25 wt.% MWCNTs.

Cancellous or spongy bone has a compressive strength and Young's modulus of 2–12 MPa and 20–500 MPa, respectively [33]. Both compressive strength and elastic modulus of the scaffolds are in the range of natural spongy bone, which made them appropriate choices for bone tissue engineering applications.

### 3.3. SEM Observations

**Figure 6:**
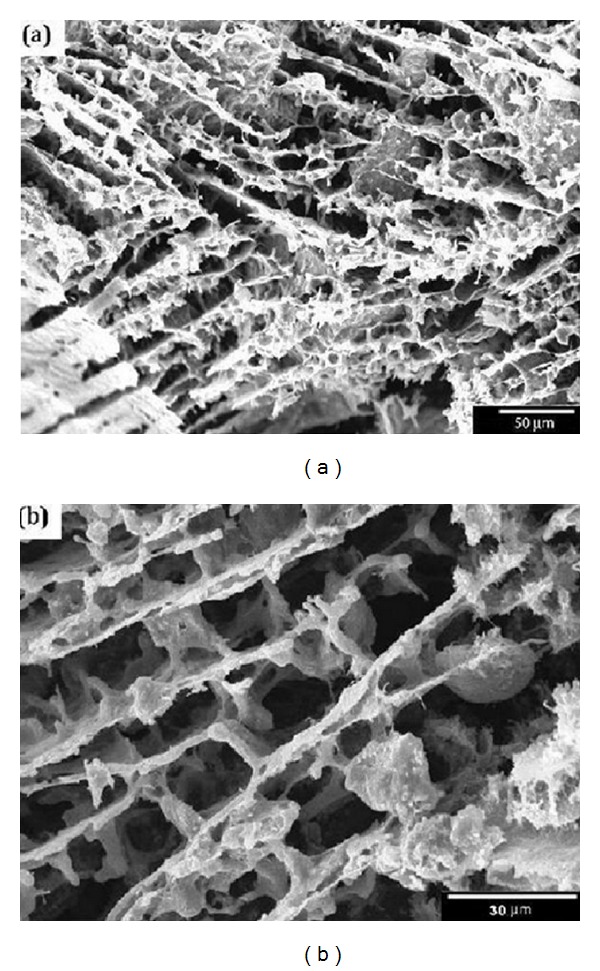
SEM micrograph of microstructure of MWCNTs/45S5 Bioglass composite scaffold with 63% porosity. (a) Cross sections parallel to the ice front, (b) with more details.

**Figure 7:**
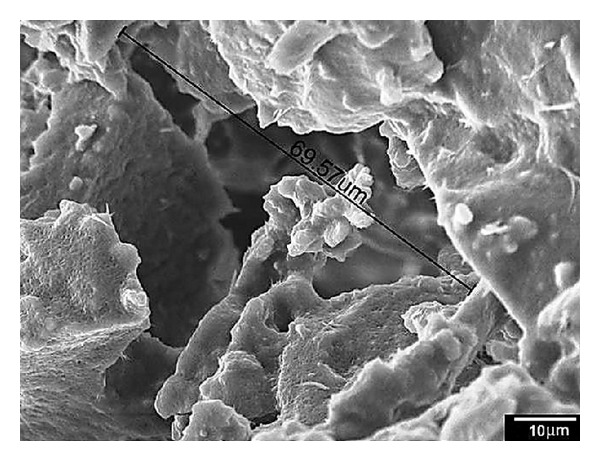
SEM micrograph of an open pore in MWCNTs/45S5 Bioglass composite scaffold.

The lamellar microstructure of the scaffolds consists of plates with flat interconnected macropores between them, aligned along the ice growth direction (Figure 6). On the internal walls of the lamellae, a dendritic, branch-like structure can be observed following the microscopic ice formation. Some ceramic bridges, linking adjacent plates, are also observed. The width of the open interconnected macropores typically ranges between 20 and 100 *μ*m, as shown in Figure 7.

**Figure 8:**
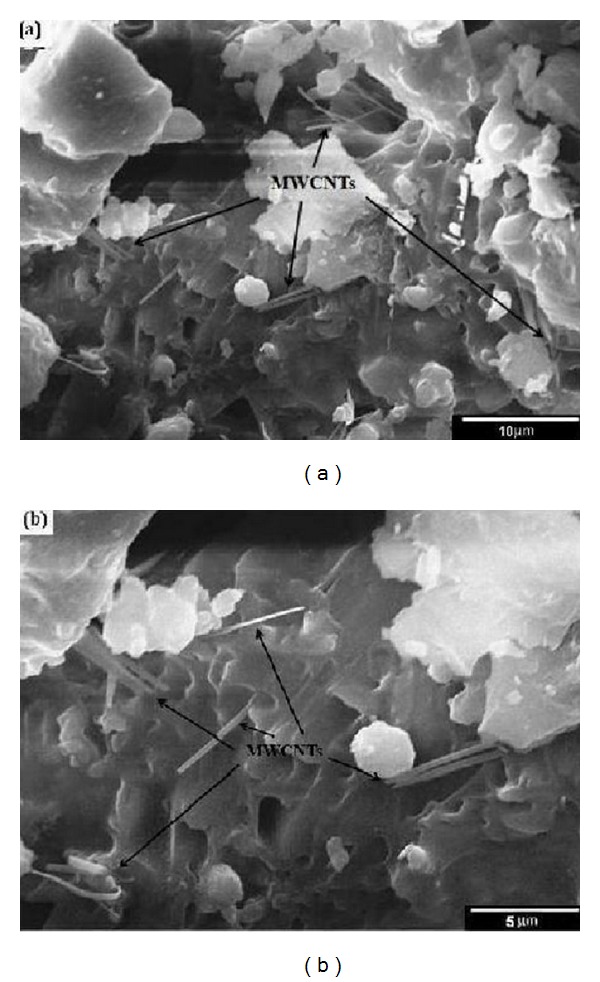
(a) Low magnification and (b) high magnification SEM micrographs of homogeneous distribution of MWCNTs in the 45S5 Bioglass matrix.

Figure 8 shows the homogeneous distribution of MWCNTs in the 45S5 Bioglass matrix in the composite scaffold in different magnifications. It is worth mentioning that CNT, as the reinforcing phase, can enhance the mechanical properties of composite only in the case of homogeneous distribution in the matrix.

**Figure 9:**
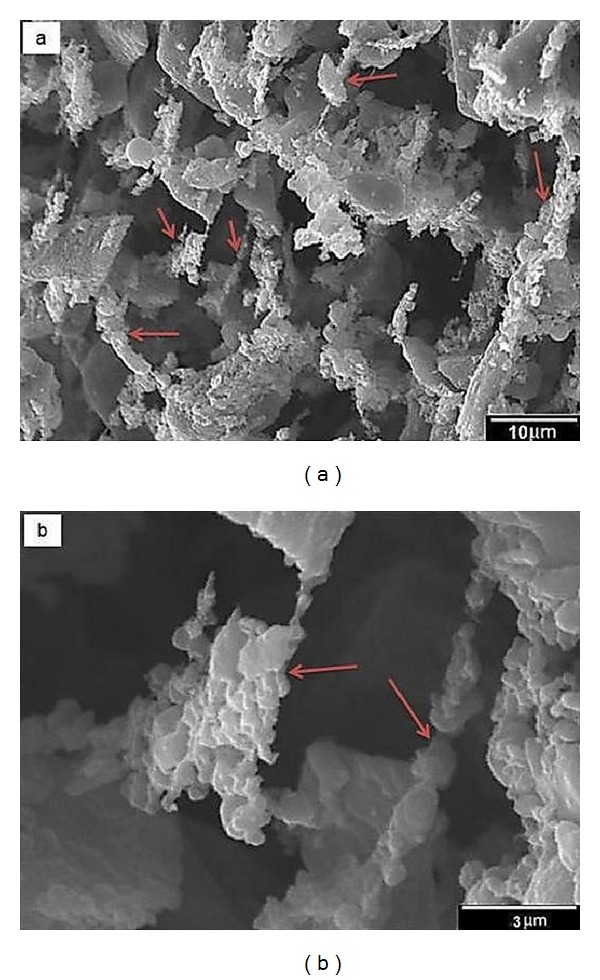
(a) Low magnification and (b) high magnification SEM micrographs of the bridges of CNTs between composite plates of the MWCNTs/45S5 Bioglass composite scaffold porosities (placing and crystallization of Bioglass particles on the CNT bridges).

As clearly shown in Figure 9, CNTs create new connections via formation of bridges between composite plates of the MWCNT/45S5 Bioglass nanocomposite scaffold porosities in different regions. It seems that this mechanism of the bridge formation plays a critical role in the improvement of the mechanical properties of these composite scaffolds used in bone tissue engineering. In addition to forming the new connections in the scaffold porosities, CNT bridges are appropriate sites for placing and crystallization of Bioglass particles.

## 4. Conclusions

Fine powders of Na_2_O-containing glass ceramics have been successfully synthesized using the sol-gel technique in aqueous solution under ambient conditions. The sol-gel, derived and sintered, 45S5 glass ceramic materials possess the essential features of Na_2_O-containing bioactive materials, namely, the formation of crystalline phase Na_2_Ca_2_Si_3_O_9_ during sintering, which couples well mechanical strength with appropriate biodegradability. MWCNT/45S5 Bioglass composite scaffolds have been successfully produced by means of the freeze-casting technique. The optimal content of MWCNTs in the composites was 0.25 wt.%. Compared to the scaffolds prepared by 100% 45S5 Bioglass powders, the addition of 0.25 wt.% MWCNTs increases the compressive strength and elastic modulus of 45S5 Bioglass scaffold from 2.08 to 4.56 MPa (a 119% increase) and 111.50 to 266.59 MPa (a 139% increase), respectively. The results have demonstrated that properties of prepared nanocomposite scaffolds were comparative to the natural spongy bone. Finally, it is important to point out that the mechanism of the bridge formation by CNTs between composite plates of scaffold porosities plays a critical role in the improvement of the mechanical properties of these composite scaffolds. The improved structural and physical properties of the MWCNT/45S5 Bioglass composites suggest their potential to be used as artificial scaffold matrix materials in bone tissue engineering.
